# rs12537 Is a Novel Susceptibility SNP Associated With Estrogen Receptor Positive Breast Cancer in Chinese Han Population

**DOI:** 10.3389/fmed.2021.708644

**Published:** 2021-07-28

**Authors:** Jingkai Xu, Guozheng Li, Mengyun Chen, Wenjing Li, Yaxing Wu, Xuejun Zhang, Yong Cui, Bo Zhang

**Affiliations:** ^1^Department of Dermatology, China-Japan Friendship Hospital, Beijing, China; ^2^Department of Dermatology, The First Affiliated Hospital of Anhui Medical University, Hefei, China; ^3^School of Life Sciences, Anhui Medical University, Hefei, China; ^4^Department of Oncology, No. 2 Hospital, Anhui Medical University, Hefei, China

**Keywords:** breast cancer, rs12537, phenotype, estrogen receptor, MTMR3

## Abstract

Genetic testing is widely used in breast cancer and has identified a lot of susceptibility genes and single nucleotide polymorphisms (SNPs). However, for many SNPs, evidence of an association with breast cancer is weak, underlying risk estimates are imprecise, and reliable subtype-specific risk estimates are not in place. A recent genome-wide long non-coding RNA (lncRNA) association study in Chinese Han has verified a genetic association between rs12537 and breast cancer. This study is aimed at investigating the association between rs12537 and the phenotype. We collected the clinical information of 5,634 breast cancer patients and 6,308 healthy controls in the early study. And χ2 test was used for the comparison between different groups in genotype. The frequency of genotypic distribution among SNP rs12537 has no statistically significant correlation with family history (*p* = 0.8945), menopausal status (*p* = 0.3245) or HER-2 (*p* = 0.2987), but it is statistically and significantly correlated with ER (*p* = 0.004006) and PR (*p* = 0.01379). Most importantly, compared to the healthy control, rs12537 variant is significantly correlated with ER positive patients and the *p*-value has reached the level of the whole genome (*p* = 1.66E-08 <5.00E-08). Furthermore, we found rs12537 associated gene *MTMR3* was lower expressed in breast cancer tissues but highly methylated. In conclusion, our findings indicate that rs12537 is a novel susceptibility gene in ER positive breast cancer in Chinese Han population and it may influence the methylation of *MTMR3*.

## Introduction

The burden of breast cancer is increasing worldwide. Among the 19.3 million new cases reported by the GLOBOCAN 2020, breast cancer patients account for 11.7% (1). China is undergoing cancer transition with an increasing burden of breast cancer, and the incidence of breast cancer arrives at 18.41%. In China, female breast cancer patients took up approximately 18% of breast cancer deaths across the world (2). Many sequencing methods such as genome-wide association studies (GWASs), exome and lncRNA sequencing are used to identify SNPs/loci/genes related to the occurrence, development, prognosis and drug resistance of breast cancer (3–7). Breast cancer is a heterogeneous and polygenic disease, and breast cancer susceptibility SNPs and genes are closely related to molecular subtype and clinical phenotypes (8–10). However, for many SNPs, evidence of an association with cancer is often weak, and accurate estimates of the cancer risks associated with variants are often not available (11). In our previous study, we performed a genome-wide lncRNA association study, and reported a suggestive SNP, rs12537 (*p* = 8.84E-07), which may be associated with breast cancer susceptibility (12). rs12537 variant was reported to be associated with IgA nephropathy in Han Chinese, and rheumatoid arthritis (RA) and systemic lupus erythematosus (SLE) in Egyptian patients (13, 14). Moreover, rs12537 variant was also found associated with significantly increased gastric cancer risk (15, 16). However, the relationship between rs12537 and breast cancer remains unknown.

To investigate the association between rs12537 on 22q12.2 and breast cancer susceptibility as well as the clinical phenotype including familial history, menopausal status, estrogen receptor (ER), progestogen receptor (PR), human epidermal growth factor receptor 2 (HER-2) and molecular subtypes of breast cancer patients in Chinese Han population, we conducted a genotype-phenotype analysis to clarify the association of rs12537 with breast cancer phenotypes in Chinese Han population. Moreover, we tried to analyze rs12537 associated genes and breast cancer based on public databases.

## Materials and Methods

### Subjects

We collected the genotyping data from our previous data (including GWAS stage data and replication stage genotyping data) and clinical data (including age of onset, family history, menopausal status, ER, PR and HER-2) of a total of 5,634 patients (12). Immunohistochemical analysis was employed to evaluate the ER, PR and HER-2 status of breast tissue of biopsies. Each case was diagnosed and confirmed by at least two oncologists. And their clinical information was collected by investigators with a comprehensive clinical check-up. We also collected the genotyping data and age of 6,308 healthy controls, and they were clinically determined to be free of breast cancer, other neoplastic disease, systemic disorders, and to have no family history of cancer (including first-, second- and third-degree relatives). All participants provided written informed consent. This study was approved by the institutional ethics committee of each hospital and was conducted according to the Declaration of Helsinki principles.

### Statistical Analysis

To identify which phenotypes were associated with the specific SNP rs12537, we performed case-control and case-only analysis to examine the risk conferred by the suggestive SNP on different phenotypes of breast cancer. PLINK1.07 software (developed by Christopher Chang and others) and SPSS16.0 (IBM, https://www.ibm.com) were used to perform chi-square test and logistic regression analysis to explore the correlation between rs12537 and breast cancer susceptibility, as well as the different phenotypes of breast cancer. Allele frequency and genotype frequency were calculated by direct counting method, χ^2^ significance test was carried out, and the relative risk was evaluated by Odds ratio (OR) and 95% confidence interval (95% CI), with the difference being statistically significant (*p* < 0.05), a remarkable deviation from Hardy-Weinberg equilibrium in the controls (*p* > 0.05) during each stage.

## Results

### Sample Characteristics

All subjects involved in this study were from our early genome-wide lncRNA association study (12). The clinical features of 5,634 cases and 6,308 controls are intact in this study. The average age of onset of 5,634 female breast cancer patients was 50.7 ± 11.1. 4.70% (265) of these patients had familial history of cancer, 50.15% (2,118) were diagnosed with premenopausal breast cancer, 65.05% (2,773) were ER positive, 63.44% (2,704) were PR positive, and 26.99% (1,148) were HER-2 positive. For molecular subtypes, data of 1,538 of these patients were missing, 26.56% (1,088), 34.55% (1,415) and 15.38% (630) were lumina A, lumina B and HER-2 amplified breast cancer carriers, respectively, and 23.51% (963) were diagnosed with basal-like breast cancer. The average age of 6,308 female healthy controls was 47.4 ± 12.8 (Table 1).

**Table 1:** Baseline characteristics of breast cancer patients and healthy controls.

**Characteristics**	**Total Samples**	**GWAS Samples**	**Replication Samples**
**Cases**			
Sample size	5,634	1,496	4,138
Mean age at onset (SD)	50.7 ± 11.1	49.3 ± 10.9	51.2 ± 11.2
Mean age (SD)	51.0 ± 11.1	50.0 ± 10.9	51.3 ± 11.1
**Familial history of cancer**			
Familial (%)	265 (4.70%)	24 (1.60%)	241 (5.82%)
Sporadic (%)	5,369 (95.30%)	1472 (98.40%)	3,897 (94.18%)
Menopausal status	4,223	849	3,374
Premenopausal (%)	2,118 (50.15%)	471 (55.48%)	1,647 (48.81%)
Postmenopausal (%)	2,105 (49.85%)	378 (44.52%)	1,727 (51.19%)
ER	4,263	703	3,560
Positive (%)	2,773 (65.05%)	456 (64.86%)	2,317 (65.08%)
Negative (%)	1,490 (34.95%)	247 (35.14%)	1,243 (34.92%)
PR	4,262	702	3,560
Positive (%)	2,704 (63.44%)	413 (58.83%)	2,291 (64.35%)
Negative (%)	1,558 (36.56%)	289 (41.17%)	1,269 (35.65%)
HER-2	4,254	698	3,556
Positive (%)	1,148 (26.99%)	136 (19.48%)	1,012 (28.46%)
Negative (%)	3,106 (73.01%)	562 (80.52%)	2,544 (71.54%)
Molecular subtypes	4,096	687	3,409
Luminal A breast cancer	1,088 (26.56%)	207 (30.13%)	881 (25.84%)
Lumina B breast cancer	1,415 (34.55%)	244 (35.52%)	1,171 (34.35%)
HER-2 amplified breast cancer	630 (15.38%)	93 (13.54%)	537 (15.75%)
Basal-like breast cancer	963 (23.51%)	143 (20.82%)	820 (24.05%)
**Controls**			
Sample size	6,308	1,257	5,051
Mean age (SD)	47.4 ± 12.8	37.2 ± 11.9	50.3 ± 10.6

### Genotypic and Phenotype Analysis

To further explore the relationship between suggestive SNP rs12537 and breast cancer susceptibility, we combined our GWAS and replication data to perform a genotypic and phenotype analysis based on the clinical information we collected. The results show that the suggestive SNP rs12537 is not related to the familial history of cancer, menopausal status, HER-2 and the four molecular subtypes of breast cancer patients. And there is a statistical difference between PR positive patients and PR negative patients (*p* = 0.01379, OR = 0.8536, 95% CI: 0.7525–0.9638) in rs12537 variant (Table 2 and Supplementary Table 1). And we also performed genotypic and phenotype analysis on the other three SNPs, rs9397435, rs11066150 and rs62112521 in Chinese Han women (12), but we found no correlation between these three SNPs and the clinical characteristics of breast cancer (Supplementary Table 2).

**Table 2:** The genotypic and allelic frequency of rs12537.

	**Groups**	**Allele frequency (%)**		***p*-value**	**OR (95% CI)**	***p*-hwe**
		**T**	**C**			
Family history	Positive vs. Control	0.175	0.205	0.1095	0.8226 (0.6475-1.045)	0.6475
	Negative vs. Control	0.1774	0.205	1.44E-06	0.8362 (0.7775–0.8994)	0.7775
	Positive vs. Negative	0.175	0.1774	0.8945	0.9837 (0.7719–1.254)	0.7719
Menopausal status	Positive vs. Control	0.1751	0.205	9.89E-05	0.8231 (0.7462–0.908)	0.7462
	Negative vs. Control	0.1843	0.205	0.008677	0.8764 (0.7941–0.9672)	0.7941
	Positive vs. Negative	0.1751	0.1843	0.3245	0.9393 (0.8292–1.064)	0.8292
ER	Positive vs. Control	0.1664	0.205	1.66E-08	0.7744 (0.7085–0.8464)	0.7085
	Negative vs. Control	0.1938	0.205	0.2052	0.9321 (0.8360–1.0390)	0.836
	Positive vs. Negative	0.1664	0.1938	0.004006	0.8309 (0.7323–0.9427)	0.7323
PR	Positive vs. Control	0.1678	0.205	6.13E-08	0.7822 (0.7155–0.8550)	0.7155
	Negative vs. Control	0.1911	0.205	0.114	0.9163 (0.8222–1.021)	0.8222
	Positive vs. Negative	0.1678	0.1911	0.01379	0.8536 (0.7525–0.9683)	0.7525
HER-2	Positive vs. Control	0.1832	0.205	0.02393	0.8699 (0.7708–0.9818)	0.7708
	Negative vs. Control	0.1728	0.205	1.14E-06	0.8101 (0.7441–0.8819)	0.7441
	Positive vs. Negative	0.1832	0.1728	0.2987	1.0740 (0.9388–1.2280)	0.9388

Surprisingly, ER positive patients and healthy controls also differ statistically (*p* = 1.66E-8, OR = 0.7744, 95% CI: 0.7085–0.8464) in rs12537 variant, and this difference has reached the level of the whole genome for *p* <5.00E-8. Moreover, there is also a statistical difference between ER positive patients and ER negative patients (*p* = 0.004006, OR = 0.8309, 95% CI: 0.7323–0.9427) in rs12537 variant (Table 2). To exclude the influence of clinical features other than ER status on the results, we further analyzed and compared the clinical differences between ER positive, ER negative breast cancer patients and healthy controls (Supplementary Table 3), and we found that age, family history and HER-2 expression were not correlated with ER. So rs12537 is a novel ER positive breast cancer associated SNP variant in Chinese Han women.

### rs12537 Associated Gene MTMR3 and Breast Cancer

Expression quantitative trait locus (eQTL) has become a common tool to interpret the regulatory mechanisms of the variants associated with complex traits through genome-wide association studies (GWAS) (17, 18). To identify rs12537 associated genes, based on the eQTLGen database (https://www.eqtlgen.org/), we identified 8 cis-eQTL effects genes (*ASCC2, NIPSNAP1, MTMR3, ZMAT5, SEC14L3, DUSP18, SF3A1* and *THOC5*) and 6 trans-eQTL effects genes (*FHIT, CCR7, EPHX2, LEF1, PAQR8* and *TRABD2A*) (Table 3).

**Table 3:** eQTL analysis to identify rs12537 associated genes.

***p*-value**	**Gene ID**	**Gene Symbol**	**Chr**	**Pos (hg19)**	**Z-score**	**FDR**
4.91E-71	ENSG00000100325	ASCC2^a^	22	30209434	−17.8204	0
1.08E-34	ENSG00000184117	NIPSNAP1^a^	22	29964061	−12.2859	0
8.72E-30	ENSG00000100330	MTMR3^a^	22	30352999	−11.3358	0
4.64E-23	ENSG00000100319	ZMAT5^a^	22	30144972	9.8893	0
1.15E-08	ENSG00000100012	SEC14L3^a^	22	30855991	−5.707	6.43E-05
6.63E-08	ENSG00000167065	DUSP18^a^	22	31055957	5.401	0.000253
1.42E-06	ENSG00000099995	SF3A1^a^	22	30740457	−4.8224	0.003981
9.10E-06	ENSG00000100296	THOC5^a^	22	29926536	4.4376	0.023729
3.85E-08	ENSG00000189283	FHIT^b^	3	60486084	5.4974	0.000499
6.03E-08	ENSG00000126353	CCR7^b^	17	38715872	5.418	0.000806
4.52E-07	ENSG00000120915	EPHX2^b^	8	27375688	5.0458	0.004067
7.11E-07	ENSG00000138795	LEF1^b^	4	109029406	4.9584	0.00614
1.28E-06	ENSG00000170915	PAQR8^b^	6	52249397	4.8428	0.010193
8.11E-06	ENSG00000186854	TRABD2A^b^	2	85091453	4.4624	0.04903

a *cis-eQTL effects genes*.

b *trans-eQTL effects genes*.

**Figure 1:**
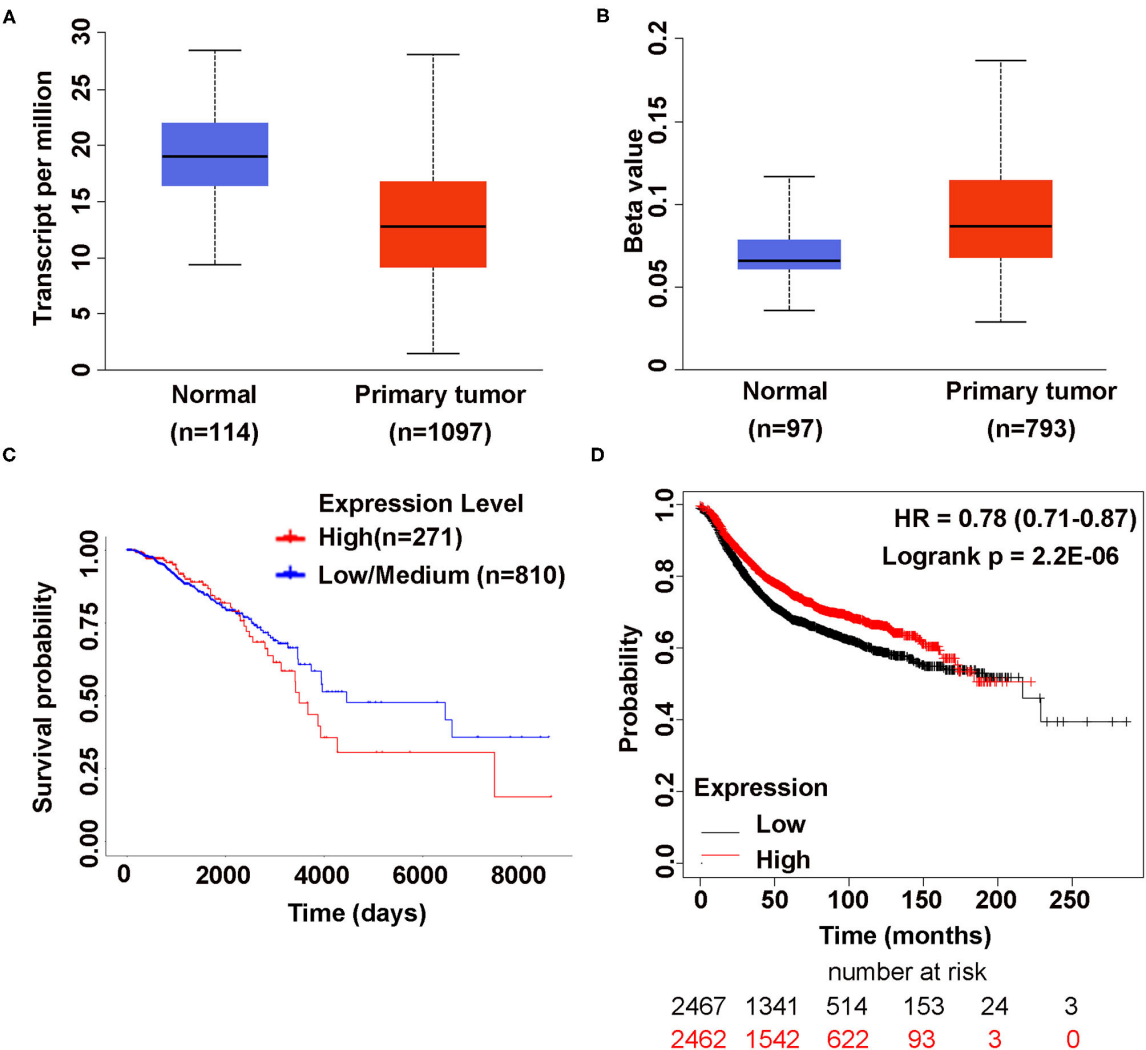
The Association Between MTMR3 Gene Expression and Breast Cancer. **(A)** MTMR3 is highly expressed in normal tissues compared to primary tumor tissues (*p* <1E-12). **(B)** MTMR3 promoter is hypermethylated in primary tumor tissues compared to normal tissues (*p* = 2.66E-02). The Beta value indicates level of DNA methylation ranging from 0 (unmethylated) to 1 (fully methylated). **(C)** MTMR3 gene expression has no correlation with the overall survival of breast cancer patients (*p* =0.44). **(D)** Lower expressed MTMR3 could reduce patient's relapse free survival (RFS) (*p* =2.2E-06) (KIAA0371 202197_at).

rs12537 associated gene, *MTMR3*, was reported to be associated with RA and SLE, gastric cancer and breast cancer (14, 15, 19). Therefore, we try to investigate the expression of *MTMR3* in the cancer genome atlas (TCGA) database by UALCAN (20), discovering that compared to normal tissues *MTMR3* was lower expressed in primary tumor tissues (*p* <1E-12), but the promoter methylation level was higher (*p* = 2.66E-02). *MTMR3* expression was not associated with overall survival (OS) (*p* = 0.44) (Figures 1A–C). Moreover, based on Kaplan-Meier Plotter (www.kmplot.com) (21), we found highly expressed *MTMR3* could improve patients relapse free survival (RFS) (*p* = 2.2E-06) (Figure 1D), but there was no correlation between *MTMR3* expression and the OS, postoperative survival (PPS) as well as distant metastasis-free survival (DMFS) in breast cancer patients (Supplementary Figure 1).

## Discussion

Breast cancer is a complex multifactorial disease, with high incidence, strong invasiveness, metastasis and heterogeneity (1, 22, 23). A large number of sequencing studies have identified more than 200 susceptibility SNPs/genes (24). By combining sequencing analysis with the clinical characteristics of breast cancer patients, more SNPs/genes that have a stronger correlation with clinical characteristics were identified, which provides important theoretical support for precision treatment of breast cancer (8, 11, 25).

It is reported that more than 60% of breast cancers, including Luminal A and Luminal B breast cancers, were ER positive (8, 10). And ER positive breast cancer is a highly heterogeneous disease comprising different histological and mutational patterns, with varied clinical courses and responses to systemic treatment. GWASs have identified a lot of ER positive breast cancer associated SNPs, such as rs112545418, rs17132398 in 4p16, rs116638271, rs77274510 and rs117564384 in 11q13 and rs10941679 in 5p12 (26, 27). In our previous study, we designed a lncRNA array independently, and then performed the first genome-wide lncRNA association study on Han Chinese women, identifying a novel breast cancer-associated susceptibility SNP, rs11066150, a previously reported SNP, rs9397435 and two suggestive SNPs rs12537 and rs62112521 (12), but our study revealed that rs11066150, rs9397435 and rs6211252 had no relationship with the clinical characteristics of breast cancer (Supplementary Table 2). In the present research, we identified rs12537 as a novel susceptibility SNP in ER positive breast cancer in Han Chinese women. And this is the first time that rs12537 has been reported to be associated with ER positive breast cancer. However, only 4,263 ER patients (65.05% ER positive, 34.56% ER negative) and 6,308 healthy controls were included in this study, and a larger and better-matched population (including age, familial history, menopausal status, ER, PR and HER-2) may be needed for further verification.

The SNP rs12537 present in the miR-181a-binding site in the 3' UTR of the *MTMR3* gene (15) and T/C variant in *MTMR3* were reported to be associated with IgA nephropathy, RA, SLE and gastric cancer (13–16). As an autophagy-related gene involved in the negative regulation of autophagy initiation (24), rs12537 T/T carriers were associated with lower serum *MTMR3* expression and higher miR-181a expression than in other genotypes among SLE patients, and their interaction may lead to autophagy increasing (14). rs12537 CT genotype carriers in gastric cancer had low *MTMR3* mRNA expression than CC genotype carriers (15). Ectopic expression of miR-181a mimics or introduction of *MTMR3* small interfering RNA resulted in an increase in cell proliferation, colony formation, migration, invasion, as well as suppression of apoptosis in gastric cancer (28).

DNA methylation plays a crucial role in the formation and process of cancers and it could be potential candidate biomarkers for cancers (29). Based on TCGA database, we found that *MTMR3* gene was lower expressed in breast cancer tissues than normal tissues and the promoter methylation level was higher. However, *MTMR3* expression had no correlation with overall survival. Here, we hypothesize that rs12537 variant in ER positive breast cancer patients could regulate the methylation of *MTMR3*, and further studies are required to fully understand the mechanism.

In conclusion, the results of our study show that rs12537 is a novel susceptibility SNP in ER positive breast cancer in Chinese Han population. Moreover, rs12537 associated gene *MTMR3* is lowly expressed but highly methylated in breast cancer. Considering that we have not found the correlation between *MTMR3* expression and overall survival based on TCGA database, multicentric studies involving a larger number of cases and genotypic data are needed to verify this result.

## Data Availability Statement

The original contributions presented in the study are included in the article/Supplementary Material, further inquiries can be directed to the corresponding authors.

## Ethics Statement

The studies involving human participants were reviewed and approved by this study was approved by the Ethics Committee of Anhui Medical University. The patients/participants provided their written informed consent to participate in this study.

## Author Contributions

JX, YC, and BZ conceived of the idea. JX, GL, and MC conducted statistical analyses. WL and YW collected clinical data. JX and GL wrote the manuscript with inputs from all authors. All authors contributed to the article and approved the submitted version.

## Conflict of Interest

The authors declare that the research was conducted in the absence of any commercial or financial relationships that could be construed as a potential conflict of interest.

## Publisher's Note

All claims expressed in this article are solely those of the authors and do not necessarily represent those of their affiliated organizations, or those of the publisher, the editors and the reviewers. Any product that may be evaluated in this article, or claim that may be made by its manufacturer, is not guaranteed or endorsed by the publisher.
