# Supplementary material for: rs12537 Is a Novel Susceptibility SNP Associated With Estrogen Receptor Positive Breast Cancer in Chinese Han Population
Source: Front Med (Lausanne). 2021 Jul 28;8:708644. doi: 10.3389/fmed.2021.708644 (PMC8355624; doi:10.3389/fmed.2021.708644)
Supplement: Supplementary file 1 [file Data_Sheet_1.docx]

Supplementary Material

# Supplementary Figures and Tables

## Supplementary Figures

**Supplementary Figure 1.** The Association Between MTMR3 Gene Expression and Breast Cancer. The expression of MTMR3 is not correlated to PPS(A), RFS (B) and DMSF (KIAA0371 202197_at).

## Supplementary Tables

**Supplementary Table 1 The genotypic and allelic frequency of rs12537 in four breast cancer molecular subtypes**

|  | **Groups** | **Allele frequency (%)** | | ***p* value** | **OR (95%CI)** | ***p*-hwe** |
| --- | --- | --- | --- | --- | --- | --- |
|  |  | **T** | **C** |  |  |  |
| **Luminal A breast cancer** | Positive VS Control | 0.1602 | 0.2050 | 1.14E-05 | 0.7397(0.6463-0.8466) | 0.6969 |
|  | Negative VS Control | 0.1784 | 0.2050 | 1.06E-04 | 0.8423(0.7722-0.9187) | 0.6969 |
|  | Positive VS Negative | 0.1602 | 0.1784 | 0.08583 | 0.8782(0.7572-1.019) | 0.994092 |
| **Lumina B breast cancer** | Positive VS Control | 0.1671 | 0.2050 | 3.79E-05 | 0.7781(0.6904-0.8770) | 0.98532 |
|  | Negative VS Control | 0.1768 | 0.2050 | 7.83E-05 | 0.8330(0.7608-0.9122) | 0.98596 |
|  | Positive VS Negative | 0.1671 | 0.1768 | 0.3287 | 0.9341(0.8146-1.071) | 0.994092 |
| **HER-2 amplified breast cancer** | Positive VS Control | 0.1926 | 0.2050 | 0.3496 | 0.9252(0.7861-1,089) | 0.98444 |
|  | Negative VS Control | 0.1699 | 0.2050 | 5.31E-08 | 0.7937(0.7302-0.8627) | 0.98659 |
|  | Positive VS Negative | 0.1926 | 0.1699 | 0.08093 | 0.7937(0.9812-1.3850) | 0.994092 |
| **Basal-like breast cancer** | Positive VS Control | 0.1862 | 0.2050 | 0.09517 | 0.8872(0.7708-1.0210) | 0.98448 |
|  | Negative VS Control | 0.1698 | 0.2050 | 1.21E-07 | 0.7933(0.7280-0.8644) | 0.98661 |
|  | Positive VS Negative | 0.1862 | 0.1698 | 0.1511 | 1.1180(0.9599-1.3030) | 0.994092 |

**Supplementary Table 2 The genotypic and allelic frequency of rs9397437, rs11066150 and rs62112521**

| **rs9397435** | **Groups** | **Allele frequency (%)** | | ***p* value** | **OR (95% CI)** | ***p*-hwe** |
| --- | --- | --- | --- | --- | --- | --- |
|  |  | G | A |  |  |  |
| **Family history** | Positive VS Control | 0.4100 | 0.2896 | 1.41E-08 | 1.705 (1.415-2.054) | 0.0964 |
|  | Negative VS Control | 0.3642 | 0.2896 | 2.50E-28 | 1.405 (1.323-1.493) | 0.0964 |
|  | Positive VS Negative | 0.4100 | 0.3642 | 0.04354 | 1.213 (1.005-1.465) | 0.0195 |
| **Menopausal status** | Positive VS Control | 0.3651 | 0.2896 | 2.44E-17 | 1.411 (1.302-1.528) | 0.0964 |
|  | Negative VS Control | 0.3651 | 0.2896 | 9.74E-17 | 1.411 (1.300-1.530) | 0.0964 |
|  | Positive VS Negative | 0.3651 | 0.3651 | 2.76E-06 | 0.999 (0.905-1.105) | 0.0414 |
| **ER** | Positive VS Control | 0.3604 | 0.2896 | 9.66E-19 | 1.382 (1.286-1.485) | 0.0964 |
|  | Negative VS Control | 0.3807 | 0.2896 | 2.77E-19 | 1.508 (1.378-1.650) | 0.0964 |
|  | Positive VS Negative | 0.3604 | 0.3807 | 0.09261 | 0.9167 (0.8282-1.015) | 0.0111 |
| **PR** | Positive VS Control | 0.3586 | 0.2896 | 8.96E-18 | 1.372 (1.276-1.474) | 0.0964 |
|  | Negative VS Control | 0.3831 | 0.2896 | 1.65E-20 | 1.523 (1.393-1.665) | 0.0964 |
|  | Positive VS Negative | 0.3586 | 0.3831 | 0.04183 | 0.9005 (0.814-0.9961) | 0.02328 |
| **HER-2** | Positive VS Control | 0.3737 | 0.2896 | 2.76E-14 | 1.464 91.327-1.616) | 0.0964 |
|  | Negative VS Control | 0.3648 | 0.2896 | 2.84E-22 | 1.409 (1.315-1.510) | 0.0964 |
|  | Positive VS Negative | 0.3737 | 0.3648 | 0.4852 | 1.039 (0.9332-1.157) | 0.05743 |
| **Luminal A breast cancer** | Positive VS Control | 0.3600 | 0.2896 | 2.07E-09 | 1.38 (1.241-1.533) | 0.0964 |
|  | Negative VS Control | 0.3713 | 0.2896 | 1.61E-24 | 1.449 (1.349-1.556) | 0.0964 |
|  | Positive VS Negative | 0.3600 | 0.3713 | 0.4046 | 0.9523 (0.8488-1.0680) | 0.009491 |
| **Lumina B breast cancer** | Positive VS Control | 0.3637 | 0.2896 | 3.21E-12 | 1.402 (1.275-1.542) | 0.0964 |
|  | Negative VS Control | 0.3706 | 0.2896 | 1.67E-22 | 1.444 (1.341-1.555) | 0.0964 |
|  | Positive VS Negative | 0.3637 | 0.3706 | 0.5881 | 0.9707 (0.8717-1.081) | 0.0426 |
| **HER-2 amplified breast cancer** | Positive VS Control | 0.3652 | 0.2896 | 4.93E-07 | 1.411 (1.233-1.615) | 0.0964 |
|  | Negative VS Control | 0.3688 | 0.2896 | 1.87E-25 | 1.433 (1.339-1.534) | 0.0964 |
|  | Positive VS Negative | 0.3652 | 0.3688 | 0.8306 | 0.9848 (0.8556-1.133) | 0.1969 |
| **Basal-like breast cancer** | Positive VS Control | 0.3874 | 0.2896 | 2.86E-14 | 1.551 (1.384-1.738) | 0.0964 |
|  | Negative VS Control | 0.3627 | 0.2896 | 6.88E-21 | 1.396 (1.302-1.497) | 0.0964 |
|  | Positive VS Negative | 0.3874 | 0.3627 | 0.08965 | 1.111 (0.9838-1.255) | 0.04015 |
| **rs11066150** | **Groups** | **Allele frequency (%)** | | ***p* value** | **OR (95% CI)** | ***p*-hwe** |
|  |  | **A** | **G** |  |  |  |
| **Family history** | Positive VS Control | 0.4391 | 0.389 | 0.028 | 1.229 (1.022-1.479) | 0.2207 |
|  | Negative VS Control | 0.4255 | 0.389 | 2.53E-06 | 1.149 (1.084-1.218) | 0.2207 |
|  | Positive VS Negative | 0.4391 | 0.4225 | 0.4777 | 1.070 (0.8878-1.289) | 0.04 |
| **Menopausal status** | Positive VS Control | 0.439 | 0.389 | 1.34E-07 | 1.229 (1.138-1.327) | 0.2207 |
|  | Negative VS Control | 0.4159 | 0.389 | 0.005374 | 1.118 (1.034-1.21) | 0.2207 |
|  | Positive VS Negative | 0.439 | 0.4159 | 0.05746 | 1.099 (0.997-1.212) | 0.5383 |
| **ER** | Positive VS Control | 0.4331 | 0.389 | 2.22E-07 | 1.2 (1.12-1.286) | 0.2207 |
|  | Negative VS Control | 0.4212 | 0.389 | 0.002812 | 1.142 (1.047-1.248) | 0.2207 |
|  | Positive VS Negative | 0.4331 | 0.4212 | 0.3374 | 1.05 (0.9505-1.159) | 0.3807 |
| **PR** | Positive VS Control | 0.4339 | 0.389 | 1.48E-07 | 1.204 (1.123-1.29) | 0.2207 |
|  | Negative VS Control | 0.4192 | 0.389 | 0.004735 | 1.134 (1.039-1.237) | 0.2207 |
|  | Positive VS Negative | 0.4339 | 0.4192 | 0.2328 | 1.062 (0.962-1.172) | 0.5622 |
| **HER-2** | Positive VS Control | 0.4385 | 0.389 | 2.81E-05 | 1.227 (1.115-1.350) | 0.2207 |
|  | Negative VS Control | 0.4252 | 0.389 | 1.05E-05 | 1.162 (1.087-1.242) | 0.2207 |
|  | Positive VS Negative | 0.4385 | 0.4252 | 0.3097 | 1.056 (0.9507-1.173) | 0.1295 |
| **Luminal A breast cancer** | Positive VS Control | 0.415 | 0.389 | 0.03841 | 1.114 (1.006-1.235) | 0.2207 |
|  | Negative VS Control | 0.435 | 0.389 | 5.57E-08 | 1.209 (1.129-1.295) | 0.2207 |
|  | Positive VS Negative | 0.415 | 0.435 | 0.1538 | 0.9215 (0.8236-1.031) | 0.4443 |
| **Lumina B breast cancer** | Positive VS Control | 0.445 | 0.389 | 8.15E-07 | 1.259 (1.149-1.38) | 0.2207 |
|  | Negative VS Control | 0.4214 | 0.389 | 0.0002331 | 1.144 (1.065-1.229) | 0.2207 |
|  | Positive VS Negative | 0.445 | 0.4214 | 0.07121 | 1.101 90.9917-1.223) | 0.8566 |
| **HER-2 amplified breast cancer** | Positive VS Control | 0.4351 | 0.389 | 0.004328 | 1.21 (1.061-1.379) | 0.2207 |
|  | Negative VS Control | 0.4285 | 0.389 | 8.53E-07 | 1.178 (1.103-1.257) | 0.2207 |
|  | Positive VS Negative | 0.4351 | 0.4285 | 0.7017 | 1.027(0.8955-1.178) | 0.3421 |
| **Basal-like breast cancer** | Positive VS Control | 0.4195 | 0.389 | 0.02596 | 1.135 (1.015-1.269) | 0.2207 |
|  | Negative VS Control | 0.4325 | 0.389 | 1.49E-07 | 1.197 (1.119-1.28) | 0.2207 |
|  | Positive VS Negative | 0.4195 | 0.4325 | 0.3854 | 0.09483 (0.8413-1.069) | 0.9671 |
| **rs62112521** | **Groups** | **Allele frequency (%)** | | ***p* value** | **OR (95% CI)** | ***p*-hwe** |
|  |  | **A** | **G** |  |  |  |
| **Family history** | Positive VS Control | 0.4265 | 0.458 | 0.1759 | 0.8801 (0.7314-1.059) | 0.9186 |
|  | Negative VS Control | 0.4308 | 0.458 | 0.1759 | 0.8801 (0.7314-1.059) | 0.9186 |
|  | Positive VS Negative | 0.4265 | 0.4308 | 0.8536 | 0.9825 (0.8148-1.185) | 0.1299 |
| **Menopausal status** | Positive VS Control | 0.4303 | 0.458 | 0.004038 | 0.8938 (0.828-0.9649) | 0.9186 |
|  | Negative VS Control | 0.4332 | 0.458 | 0.01179 | 0.9047 (0.8368-0.978) | 0.9186 |
|  | Positive VS Negative | 0.4303 | 0.4332 | 0.808 | 0.988 (0.8965-1.089) | 0.1717 |
| **ER** | Positive VS Control | 0.4289 | 0.458 | 0.0007806 | 0.8891 (0.8301-0.9522) | 0.9186 |
|  | Negative VS Control | 0.4334 | 0.458 | 0.02556 | 0.9055 (0.8299-0.988) | 0.9186 |
|  | Positive VS Negative | 0.4289 | 0.4334 | 0.717 | 0.9819 (0.8893-1.084) | 0.5619 |
| **PR** | Positive VS Control | 0.4282 | 0.458 | 0.0005872 | 0.8861 (0.8272-0.9494) | 0.9186 |
|  | Negative VS Control | 0.4348 | 0.458 | 0.03318 | 0.9104 (0.8351-0.9926) | 0.9186 |
|  | Positive VS Negative | 0.4282 | 0.4348 | 0.5922 | 0.9734 (0.882-1.074) | 0.566 |
| **HER-2** | Positive VS Control | 0.4364 | 0.458 | 0.07227 | 0.9164 (0.8331-1.008) | 0.9186 |
|  | Negative VS Control | 0.4277 | 0.458 | 0.0002818 | 0.8845 (0.8278-0.9451) | 0.9186 |
|  | Positive VS Negative | 0.4364 | 0.4277 | 0.5075 | 1.036 (0.933-1.15) | 0.9674 |
| **Luminal A breast cancer** | Positive VS Control | 0.4118 | 0.458 | 0.0003114 | 0.8288 (0.7482-0.9179) | 0.9186 |
|  | Negative VS Control | 0.4345 | 0.458 | 0.006242 | 0.9092 (0.8493-0.9734) | 0.9186 |
|  | Positive VS Negative | 0.4118 | 0.4345 | 0.1056 | 0.9115 (0.8147-1.02) | 0.8986 |
| **Lumina B breast cancer** | Positive VS Control | 0.4314 | 0.458 | 0.02137 | 0.8979 (0.8193-0.9842) | 0.9186 |
|  | Negative VS Control | 0.4266 | 0.458 | 0.0004567 | 0.8807 (0.8203-0.9456) | 0.9186 |
|  | Positive VS Negative | 0.4313 | 0.4266 | 0.7168 | 1.02 (0.9182-1.132) | 0.7529 |
| **HER-2 amplified breast cancer** | Positive VS Control | 0.4366 | 0.458 | 0.1937 | 0.9173 (0.8053-1.045) | 0.9156 |
|  | Negative VS Control | 0.4267 | 0.458 | 0.0001263 | 0.881 (0.8257-0.94) | 0.9186 |
|  | Positive VS Negative | 0.4366 | 0.4267 | 0.5626 | 1.041 (0.9082-1.194) | 0.4518 |
| **Basal-like breast cancer** | Positive VS Control | 0.4377 | 0.458 | 0.1471 | 0.9212 (0.8245-1.029) | 0.9186 |
|  | Negative VS Control | 0.4256 | 0.458 | 0.000113 | 0.8769 (0.8203-0.9374) | 0.9186 |
|  | Positive VS Negative | 0.4377 | 0.4256 | 0.4179 | 1.051 (0.9323-1.184) | 0.409 |

**Supplementary Table 3 Baseline characteristics of ER positive breast cancer, ER negative breast cancer patients and healthy controls**

| **Characteristics** | **ER positive** | **ER negative** | **Controls** |
| --- | --- | --- | --- |
| Sample size | 2,773 | 1,490 | 6,308 |
| Mean age at onset (SD) | 51.3±11.3 | 50.4±11.0 | NA^a^ |
| Mean age (SD) | 51.6±11.3 | 50.8±11.0 | 47.4±12.8 |
| Familial history of cancer |  |  |  |
| Familial (%) | 171 (6.17%) | 83 (5.57%) | 0 |
| Sporadic (%) | 2,602 (93.83%) | 1,407 (94.43%) | NA^a^ |
| Menopausal status | 2,649 | 1,350 |  |
| Premenopausal (%) | 1,345 (50.77%) | 647 (47.93%) | NA^a^ |
| Postmenopausal (%) | 1,304 (49.23%) | 703 (52.07%) | NA^a^ |
| PR | 2,771 | 1,490 |  |
| Positive (%) | 2,680 (96.72%) | 24 (1.61%) | NA^a^ |
| Negative (%) | 91 (3.28%) | 1,466 (98.39%) | NA^a^ |
| HER-2 | 4,254 | 1,489 |  |
| Positive (%) | 1,148 (26.99%) | 509 (34.18%) | NA^a^ |
| Negative (%) | 3,106 (73.01%) | 980 (65.82%) | NA^a^ |
| Molecular subtypes |  |  |  |
| Luminal A breast cancer | 1,083 (39.06%) | 5 (0.34%) | NA^a^ |
| Lumina B breast cancer | 1,408 (50.78%) | 7 (0.47%) | NA^a^ |
| HER-2 amplified breast cancer | 130 (4.69%) | 500 (33.56%) | NA^a^ |
| Basal-like breast cancer | 0 (0%) | 963 (64.63%) | NA^a^ |
| Unknown | 152 (5.48%) | 15 (1.01%) | NA^a^ |

^a^Unknown data
