# Supplementary figures and images for: rs12537 Is a Novel Susceptibility SNP Associated With Estrogen Receptor Positive Breast Cancer in Chinese Han Population
Source: Front Med (Lausanne). 2021 Jul 28;8:708644. doi: 10.3389/fmed.2021.708644 (PMC8355624; doi:10.3389/fmed.2021.708644)

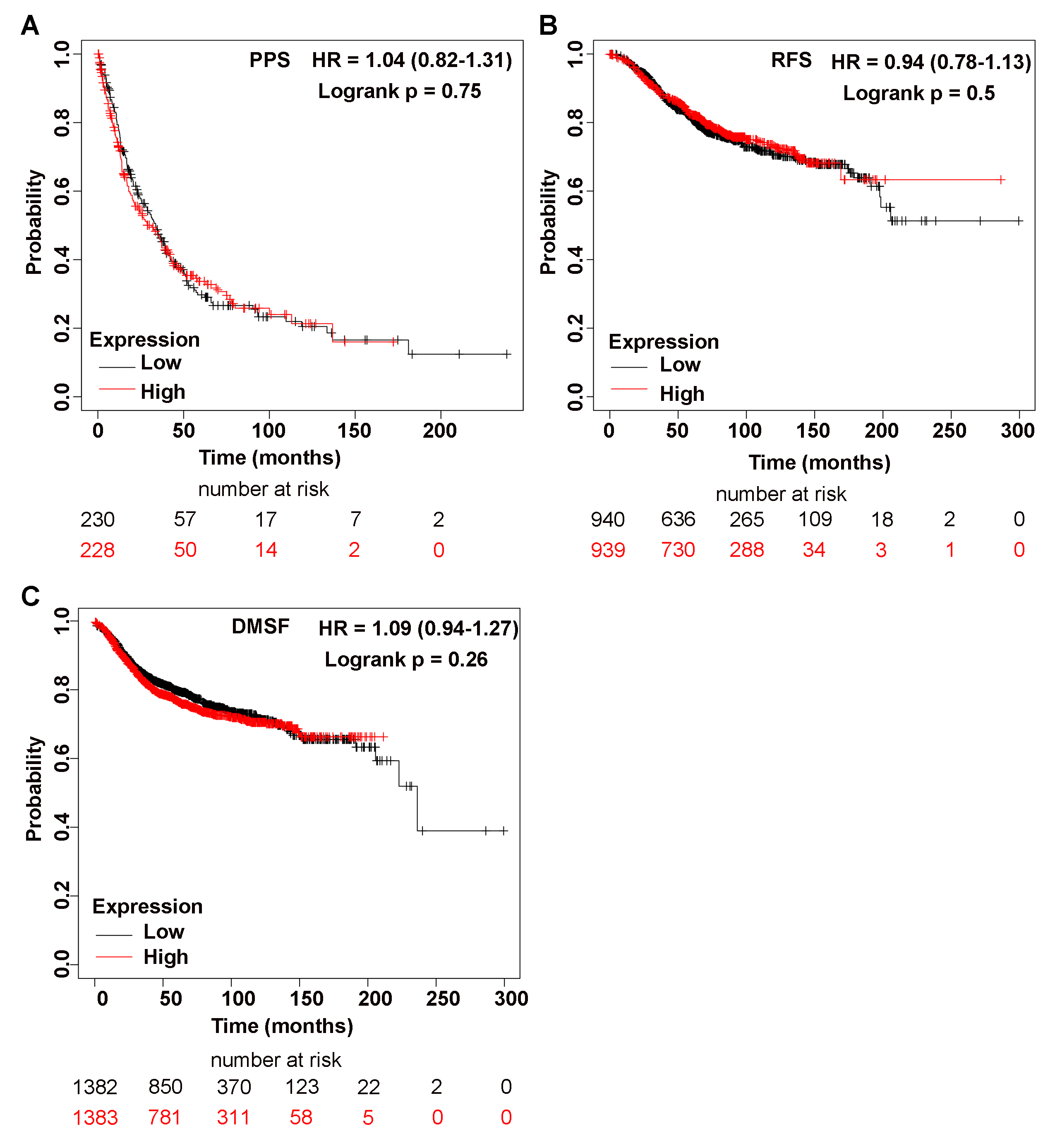

Supplement: Supplementary file 2 [file Image_1.TIF]
